# Multimodality imaging for early detection of subacute myocardial rupture

**DOI:** 10.1093/ehjcr/ytag669

**Published:** 2026-09-23

**Authors:** Giacomo Boi, Maria Francesca Marchetti, Riccardo Cau, Roberta Montisci

**Affiliations:** Department of Medical Sciences and Public Health, Cardiology Unit, University Hospital ‘Duilio Casula’—Azienda Ospedaliero-Universitaria di Cagliari, University of Cagliari, SS 554 4.5 km, Cagliari 09042, Italy; Department of Medical Sciences and Public Health, Cardiology Unit, University Hospital ‘Duilio Casula’—Azienda Ospedaliero-Universitaria di Cagliari, University of Cagliari, SS 554 4.5 km, Cagliari 09042, Italy; Department of Radiology, Azienda Ospedaliero Universitaria di Cagliari, University of Cagliari, SS 554 4.5 km, Monserrato 09042, CA, Italy; Department of Medical Sciences and Public Health, Cardiology Unit, University Hospital ‘Duilio Casula’—Azienda Ospedaliero-Universitaria di Cagliari, University of Cagliari, SS 554 4.5 km, Cagliari 09042, Italy

## Case description

A 54-year-old man with a previous anterior myocardial infarction (MI) presented with pericarditic chest pain lasting >24 h. Three days before presentation, he also reported an episode of prolonged chest pain that resolved spontaneously. Electrocardiography showed inferior ST-segment elevation. Urgent coronary angiography revealed complete occlusion of the posterolateral branch of a dominant right coronary artery, with patency of a previously implanted left anterior descending artery stent (Video 1).

The angiographic appearance did not allow definitive determination of the age of the occlusion. However, the prolonged chest pain with predominantly pericarditic features, the absence of pathological Q waves, and the lack of ongoing ischaemic symptoms at the time of angiography, raised diagnostic uncertainty between acute myopericarditis with an incidental coronary occlusion and subacute MI. Immediate revascularization was therefore deferred pending further diagnostic evaluation.

The patient was admitted to the cardiac intensive care unit, remaining haemodynamically stable. Troponin I was 8965 ng/L (reference range <57 ng/L). Transthoracic echocardiography demonstrated mildly reduced left ventricular systolic function with inferior and inferolateral akinesia and a mild circumferential pericardial effusion containing spontaneous echo contrast, suggestive of haemopericardium (*Figure 1A*). Cardiovascular magnetic resonance (CMR) was subsequently performed to clarify the underlying diagnosis after coronary angiography. It confirmed transmural oedema and late gadolinium enhancementinvolving the inferior and inferolateral walls and identified a contained rupture communicating with the left ventricular cavity (*Figure 1B* and *C*, Video 2). The pericardial effusion appeared hyperintense on T1-weighted images, consistent with haemopericardium, thereby redirecting management towards urgent surgical repair rather than coronary revascularization. ECG-gated cardiac computed tomography (CT), performed before surgery, further characterized the serpiginous myocardial tear, demonstrating a 5-mm neck and a longitudinal extension of 25 mm (*Figure 1D*, Video 3). The patient subsequently underwent successful surgical patch repair.

**Figure 1:**
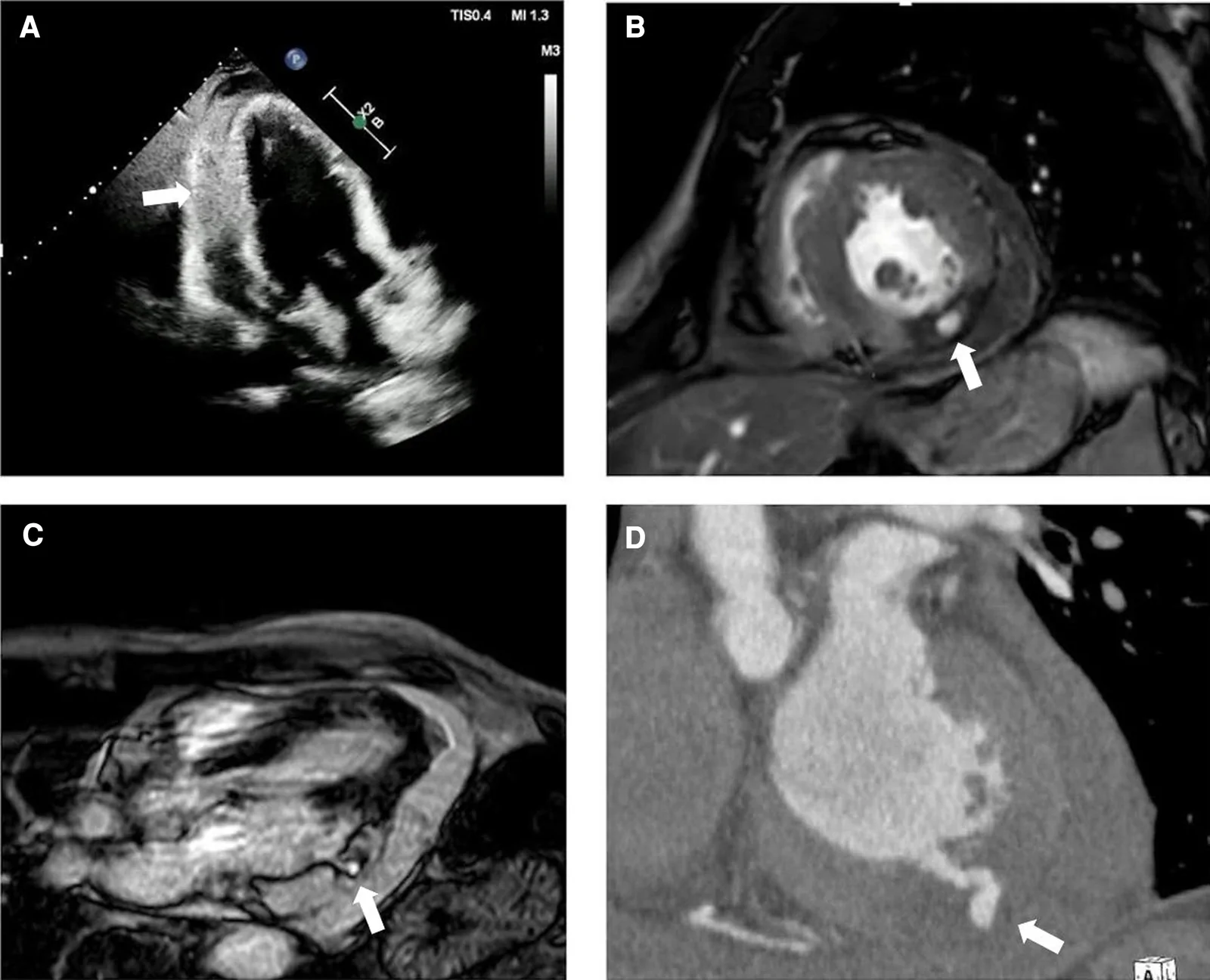
(*A*) Transthoracic echocardiographic three-chamber view demonstrating mild pericardial effusion with spontaneous echo contrast, suggestive of haemopericardium (white arrow, panel *A*). (*B*) Short-axis cine CMR image demonstrating a left ventricular pseudoaneurysm involving the mid-inferior wall (white arrow, panel *B*). (*C*) Late gadolinium enhancement CMR image showing transmural enhancement of the mid-basal inferior wall encompassing the pseudoaneurysm (white arrow, panel *C*). (*D*) ECG-gated cardiac CT demonstrating a serpiginous inferolateral left ventricular pseudoaneurysm (white arrow, panel *D*).

Left ventricular free-wall rupture is a rare but catastrophic complication of acute MI. When contained by the pericardium or organized thrombus, it may evolve into a ventricular pseudoaneurysm with a substantial risk of rupture and death if left untreated.^1^ The differential diagnosis includes contained free-wall rupture, ventricular pseudoaneurysm, and ventricular diverticulum. In contrast to ventricular diverticulum, which is usually congenital, contained rupture is typically associated with MI and haemopericardium.^2^ This case highlights the value of multimodality imaging: Echocardiography raised suspicion for haemopericardium, CMR established the diagnosis, and CT provided complementary anatomical characterization of the myocardial tear and valuable information for surgical planning, enabling prompt treatment.^1,3^

## Data Availability

The data underlying this article will be shared on reasonable request to the corresponding author.
